# A pragmatic randomized controlled trial of rapid on-site influenza and respiratory syncytial virus PCR testing in paediatric and adult populations

**DOI:** 10.1186/s12879-022-07796-3

**Published:** 2022-11-16

**Authors:** Helen L. Bibby, Lawrence de Koning, Isolde Seiden-Long, Nathan Zelyas, Deirdre L. Church, Byron M. Berenger

**Affiliations:** 1 Department of Pathology and Laboratory Medicine, University of Calgary, 3535 Research Rd NW, Calgary, AB T2L 2K8 Canada; 2 Alberta Precision Laboratories, Calgary, AB Canada; 3 Department of Laboratory Medicine and Pathology, University of Alberta, Edmonton, AB Canada

**Keywords:** Point-of-care, Rapid molecular testing, Respiratory viruses, Respiratory infection, Length of stay, Antiviral utilization, Adults, Paediatrics

## Abstract

**Background:**

Rapid/point-of-care respiratory virus nucleic acid tests (NAT) may improve oseltamivir, antibiotic, diagnostic test, and hospital bed utilization. Previous randomized controlled trials (RCT) on this topic have not used standard procedures of an accredited healthcare and laboratory system.

**Methods:**

We conducted a parallel RCT at two hospitals [paediatric = Alberta Children’s Hospital (ACH); primarily adult = Peter Lougheed Centre (PLC)]. Patients with a respiratory viral testing order were randomized to testing at either a central accredited laboratory (standard arm) or with a rapid polymerase chain reaction test at an on-site accredited laboratory followed by standard testing [rapid on-site test (ROST) arm] based on day of specimen receipt at the laboratory. Patients and clinicians were blinded to assignment.

The primary outcome for ACH was inpatient length of stay (LOS) and for PLC was the proportion of inpatients prescribed oseltamivir.

**Results:**

706 patient encounters were included at ACH; 322 assigned to ROST (181 inpatients) and 384 to the standard arm (194 inpatients). 422 patient encounters were included at PLC; 200 assigned to ROST (157 inpatients) and 222 to the standard arm (175 inpatients).

The rate of oseltamivir prescription and number of doses given was reduced in PLC inpatients negative for influenza in the ROST arm compared to standard arm [mean 14.9% (95% CI 9.87–21.9) vs. 27.5% (21.0–35.2), p = 0.0135; mean 2.85 doses (SEM 2.39–3.32) vs. 4.17 doses (3.85–4.49) p = 0.022, respectively]. ROST also significantly reduced oseltamivir use at ACH, reduced chest radiographs (ACH), and laboratory test ordering (PLC), but not antibiotic prescriptions. ROST also reduced the median turnaround time by > 24 h (ACH and PLC).

The LOS at ACH was not significantly different between the ROST and standard arms [median 4.05 days (SEM 1.79–18.2) vs 4.89 days (2.07–22.9), p = 0.062, respectively].

No adverse events were reported.

**Conclusions:**

In a RCT representing implementation of ROST in an accredited laboratory system, we found that a ROST improved oseltamivir utilization and is the first RCT to show reduced ancillary testing in both paediatric and adult populations. A larger study is required to assess reduction in paediatric LOS as ACH was underpowered. These findings help justify the implementation of rapid on-site respiratory virus testing for inpatients.

*Trial registration* ISRCTN, number 10110119, Retrospectively Registered, 01/12/2021.

**Supplementary Information:**

The online version contains supplementary material available at 10.1186/s12879-022-07796-3.

## Background

Globally, respiratory viral disease causes substantial morbidity and mortality with significant economic impact [1]. Prior to SARS-CoV-2, influenza virus and respiratory syncytial virus (RSV) were the respiratory pathogens most commonly responsible for severe disease causing hospitalization [2]. Diagnosis of these pathogens remains important to guide clinical management, to detect and manage outbreaks, for public health surveillance, and for pandemic preparedness.

The recommended method to diagnose influenza and other respiratory virus infections is by nucleic acid tests (NATs) because of their superior analytical performance compared to antigen-based tests or culture [3]. These tests have the capacity to be rapid, which is loosely defined as being able to provide results within 2 h. Some rapid NAT platforms can also be performed at the point-of-care or near to the patient. In contrast, standard NAT done within the laboratory (often at centralized off-site laboratories) have longer turnaround times (TAT) partly due to logistical considerations including specimen transport [4].

Reviews of the literature conclude that the clinical impact of rapid respiratory virus NATs is variable, reflecting the heterogeneity in quality and study design of previously reported studies [4–8]. Two of the most consistent findings are decreased TAT and improved oseltamivir utilization demonstrated by reduced time to first dose and/or increased or reduced prescription in influenza positive or negative patients, respectively [5, 8]. One randomized trial has also demonstrated decreased duration of hospitalization [9]. The Infectious Disease Society of America states that rapid NATs have the “potential to reduce unnecessary antibiotic use, improve antiviral prescribing, limit additional ancillary testing, shorten hospital or emergency department (ED) lengths of stay, and optimize infection-control practices” [6]. The American Academy of Pediatrics (AAP) also endorses the use of rapid influenza tests for similar reasons [10].

Most published studies are observational studies comparing outcomes pre and post-implementation, therefore more randomized controlled trials (RCTs) are needed [4–6, 11].

An important limitation to the majority of prior RCTs of rapid respiratory NATs is their processes do not meet laboratory “best practice” thereby limiting translation of their results outside the research environment. Collection and testing were either performed by research staff with qualifications frequently not mentioned, or the collector and test performer omitted [5, 8, 12–14]. In contrast to research settings, the implementation of patient testing occurs in the confines of accredited laboratory or health care systems with testing performed by clinical laboratory staff or health care providers. In short, truly pragmatic studies within this context are desperately needed to fully address the impact of rapid respiratory NATs when implemented in a health care system. A notable exception is a study run by Bouzid et al., on emergency department (ED) adult patients were randomized by the week of testing and providers were not blinded to the arm [15]. To address these gaps and lack of standardization adhering to laboratory or health care system “best practice” in published trials, we performed a pragmatic RCT comparing testing with a rapid on-site Flu/RSV NAT (ROST) performed in hospital laboratories to testing with a respiratory pathogen panel NAT (RPP) in the centralized laboratory. The primary outcomes of this study for inpatients were length of stay (LOS) and oseltamivir utilization. Secondary outcomes included the utilization of antibiotics, chest radiographs (CXR), and other laboratory tests.

## Methods

### Design, participants, and interventions

We conducted a parallel RCT on ED patients and inpatients (IP) who had a respiratory virus NAT ordered on a collected nasopharyngeal (NP) swab or NP aspirate (NPA) at the Alberta Children’s Hospital (ACH), a 141 bed paediatric regional tertiary hospital, and the Peter Lougheed Centre (PLC), a 522 bed tertiary hospital providing predominantly adult care, in Calgary, Alberta, Canada. This study was reviewed and approved by the University of Calgary Health Research Ethics Board, Calgary, AB (#REB19-2047).

Individuals were randomized based on the day of specimen receipt at the hospital laboratory to one of two arms: (1) standard of care (standard arm) testing where all respiratory virus test orders were performed at the centralized off-site laboratory using the NxTAG® RPP (RPP; Luminex, Austin, TX) or (2) on-site rapid testing (ROST) using the Xpert® Xpress Flu/RSV (Cepheid, Sunnyvale, CA) (ROST arm) performed by hospital-based trained and certified laboratory technicians. As not all viral pathogens are tested by the ROST, samples tested on the Xpert were also tested by the RPP at the centralized laboratory so that patients randomized to ROST received standard of care. Two separate reports (one for the Xpert and one for RPP) were generated in the ROST arm and discrepant results were reported as tested. Results were reported via the hospital and provincial electronic health record systems. Positive results were phoned to the primary clinician or unit. Participants were randomized to each arm on alternating days, starting with the standard arm on day one. Ordering clinicians were blinded to the randomization plan.

This study was retrospectively registered with ISRCTN, number 10110119. Study protocol is available as a supplementary file to this manuscript online or through the corresponding author.

### Outcomes

Primary outcomes were chosen to focus on clinical impact only. Length of stay (LOS) was the initi﻿al primary outcome selected, defined as the time from admission to the ED or direct to an inpatient unit to discharge. Due to different patient populations at each study site, it was deemed that this would not be an appropriate primary outcome at PLC (see section “Sample size determination”). Oseltamivir use was subsequently selected for PLC as the primary outcome, however this was not a good candidate at ACH because of a low oseltamivir prescription rate for those with a respiratory virus test order in the years preceding the trial (6% at ACH and 25% at PLC). Due to the clinical importance of both these outcomes, they were included as a secondary outcome for sites where they were not a primary one.

Upon review, original secondary outcomes per study protocol were modified to address the most clinically relevant measures within the scope of the data collected. Secondary outcomes included: the duration of ED visit, inpatient LOS (for PLC), oseltamivir prescription rates (for ED and ACH inpatients), number of oseltamivir doses given, the time from the patient’s first encounter to receipt of their first oseltamivir dose, the TAT for each test (i.e., time from specimen collection to reporting of results), the number of CXR(s) and ancillary laboratory tests ordered, and the prescription of bacterial antimicrobial agents during the patient’s encounter. Ancillary laboratory tests included in our analyses were those commonly ordered to work-up an ‘influenza-like illness’ (Additional file 1: Additional methods). Bacterial antimicrobial outcomes were simplified to binary outcome (prescription vs. no prescription) given complexity of the data set. Orders for CXRs, ancillary tests, and antimicrobial agents were analysed from the time of the NAT order to 24 h after the RPP was resulted. This period was modified from original protocol as we deemed this period most influenced by respiratory NAT results. Co-morbidity, mortality, and isolation data were not analysed as it was not reliably captured by our data sources.

Both primary and secondary outcomes were assessed after completion of enrolment.

### Sample size determination

The sample size required for LOS was calculated using the median duration of hospitalization from May 2017 to August 2019 for patients who had a RPP test performed (4.18 days at ACH and 6.89 days at PLC). As such, for ACH 215 inpatients per arm would be needed to detect a difference in LOS by 1 day and 640 at PLC (beta = 0.8, alpha = 0.05) [12]. The sample size required to assess LOS at PLC was therefore not attainable and the primary outcome of reducing oseltamivir use in influenza negative patients was chosen. Therefore, to detect a 10% difference in the number of patients with an oseltamivir order tested by RPP at PLC (baseline 22%), 118 patients would need to be recruited (beta = 0.8, alpha = 0.05) [12]. Based on the previous RPP test volumes, 60 days would be a sufficient recruitment period at both sites.

### Collection and testing of samples

NP swabs were collected using the Flexible Mini-tip FloqSwab in 3 mL of universal transport media (UTM-RT®; Copan S.P.A, Bresica, Italy). NP and NP aspirates (NPA) were obtained by registered nurses, physicians or respiratory therapists trained in NP/NPA collection. Rapid testing on the Xpert was performed as per manufacturer’s instructions with the aim for a TAT of < 2 h. For testing of NPA not collected in UTM-RT, 500 µL of NPA was added to 2.5 mL of UTM-RT. See Additional file 1: Table S1 for NPA validation.

### Data analysis

Outcome data was obtained from the hospital clinical and laboratory information systems [Sunrise Clinical Manager (Allscripts Healthcare Solutions Inc., Chicago, Ill); (Millennium, Cerner Corp., North Kansas City, MO) respectively] from January 6 to June 16, 2020 inclusive.

For patients who received multiple respiratory virus test orders during an admission, the first order was used to determine eligibility for randomization. Patients tested while in the ED who were admitted to hospital were counted as inpatients. Patients with an encounter starting before the study period were excluded. Oseltamivir, CXR, ancillary laboratory tests and antimicrobial utilization were sub-analysed according to positivity of test result (i.e., positive for influenza A/B, RSV, or negative for all targets). An individual was considered to of had an oseltamivir prescription if a dose was given. After trial commencement, we decided to exclude patients positive for multiple targets from secondary outcome analyses given our binary categorization (i.e., influenza A/B or RSV positive). This was not accounted for a priori*.*

Indeterminate results on the Xpert assay were treated as positive for the analysis if the RPP was positive for the relevant target.

Statistical analysis was done using Prism8, version 8.4.3 (416)(GraphPad Software, San Diego, CA). Fisher’s exact test was used for categorical variables and unpaired t-test or Mann–Whitney U test for continuous variables. Survival analysis was done using the log-rank (Mantel–Cox) test and hazard ratios determined with the log-rank approach. P-values of < 0.050 were considered statistically significant.

## Results

### Patient enrolment and characteristics

**Fig. 1:**
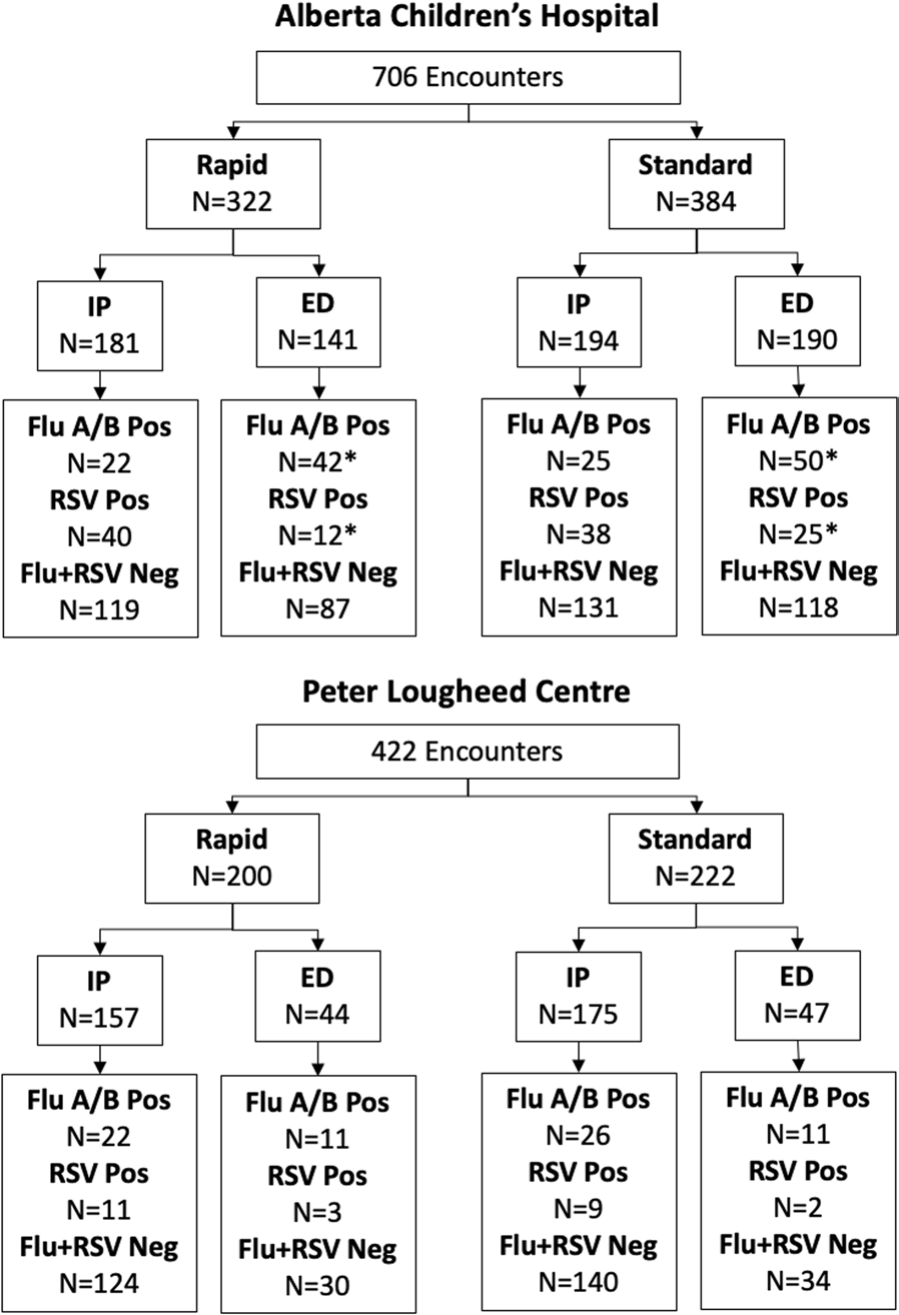
Participant flow diagram by encounter. Four ED patients at Alberta Children’s Hospital were positive for multiple targets. In the standard arm, one patient tested positive for influenza A, influenza B and RSV; two patients tested positive for influenza A and RSV. In the ROST arm, one patient tested positive for influenza A and RSV on the RPP, however tested positive for influenza A only on the ROST

A total of 706 patient encounters were enrolled at ACH; 384 (194 inpatients and 190 ED patients) were randomized into the standard arm while 322 (181 inpatients and 141 ED patients) were randomized into the ROST arm (Fig. 1). Age and sex were not different between both study arms (Table 1). The ACH study ran from January 6 to March 14, 2020 (68 days). We ran the ACH study longer than the initial 60 days attempting to recruit at least 215 patients in each arm. A long enough duration to meet this number of participants was not possible due to contractual obligations prohibiting testing on the instruments past this point.

**Table 1:** Patient demographics

	ACH All patient types^a^			ACH inpatient		
	ROST n = 322	Standard n = 384	p value	ROST n = 181	Standard n = 194	p value
Male	192 (60.6)	215 (64.0)	0.359	104 (67.5)	109 (66.2)	0.835
Female	130 (40.4)	169 (44.0)		77 (42.5)	85 (43.8)	
< 1 years	23 (7.14)	26 (6.77)	0.532	20 (11.1)	19 (9.79)	0.310
1–5 years	195 (60.6)	224 (58.3)		107 (59.1)	104 (53.6)	
6–18 years	104 (32.3)	134 (34.9)		104 (57.5)	133 (68.6)	

	PLC all patient types^a^			PLC inpatient		
	ROST n = 200	Standard n = 222		ROST n = 157	Standard n = 175	
Male	96 (48.0)	117 (52.7)	0.380	74 (47.1)	97 (55.4)	0.153
Female	104 (52.0)	105 (47.3)		83 (52.9)	78 (44.6)	
0–5 years	8 (4.00)	5 (2.25)	0.692	5 (3.18)	5 (2.86)	0.979
6–17 years	4 (2.00)	3 (1.35)		0	0	
18–65 years	93 (46.5)	109 (49.1)		65 (41.4)	73 (41.7)	
> 65 years	95 (47.5)	105 (47.3)		86 (54.8)	98 (56.0)	

Data are absolute numbers (% of total for the arm)

*ROST*=rapid on-site respiratory virus testing

^a^At ACH all encounters included 674 individuals and at PLC 408 individuals. Statistics for sex was done using Fisher’s exact test and for age chi-squared test

In the PLC study there were 422 encounters, 200 (157 inpatients and 43 ED patients) in the ROST arm and 222 (175 inpatients and 47 ED patients) in the standard arm (Fig. 1). Enrolment numbers did not match those pre-specified given this data was unavailable to study investigators during the study. The PLC study ran from January 6 to March 9, 2020 (63 days). Age and sex distribution were similar for both arms (Table 1). Analysis was by original assigned groups and no exploratory analysis was performed.

During this time period, the first case of SARS-CoV-2 reported in the Calgary health zone was on February 29, 2020. There were 62 cases identified in the Calgary health zone by the end of the enrolment period, 8 were < 18 years old. The study ran during peak influenza season (Additional file 1: Fig. S1).

### Discrepant results

In the ACH study, there was a total of nine discrepant results. In the PLC study, there was a total of 3 discrepant results (Additional file 1: Table S2).

### Primary and secondary outcomes

#### Length of stay

In the ACH study (primary outcome), the median LOS was lower in the ROST arm however did not reach statistical significance (4.05 vs 4.89 days, p = 0.062) (Additional file 1: Fig. S2). Similarly, there was no significant difference in LOS for the PLC study (secondary outcome), or for ED visit duration at either site.

#### Oseltamivir utilization

Oseltamivir utilization was improved in patients randomized to the ROST arm at both sites. In the PLC study (primary outcome) the rate of oseltamivir prescription and number of doses given was reduced in patients that were negative for influenza (Table 2). In the ACH study, there was also a significant reduction in number of doses given to influenza-negative patients (secondary outcome). The mean time to first dose of oseltamivir for patients with influenza A or B was 17.7 h shorter in the ROST arm. There were low numbers of oseltamivir prescriptions in ED patients, and outpatient prescriptions were not available (Additional file 1: Table S3). Timing of oseltamivir prescription was assessed within the ROST arm. Overall, for ACH and PLC inpatients, 61.9% and 68.4% of prescriptions, respectively, occurred after the ROST result was reported (Additional file 1: Table S4).

**Table 2:** Oseltamivir utilization for inpatients

Alberta Children’s Hospital			
	ROST	Standard	p value
Percent with oseltamivir prescription^a^			
All results	11.6 (7.72–17.1)	24.7 (19.2–31.3)	0.0013
Flu positive	68.2 (47.3–83.6)	76.0 (56.6–88.5)	0.75
Flu negative	3.77 (1.74–7.99)	17.2 (12.2–23.6)	< 0.0001
Mean number of doses^b^			
All results	4.29 (3.61–4.97)	4.97 (5.02–6.60)	0.15
Flu positive	5.33 (4.54–6.13)	8.79 (7.07–10.5)	0.079
Flu negative	1.67 (1.33–2.00)	3.72 (3.36–4.09)	0.0005
Mean hours from admission to first dose^b^			
All results	14.1 (11.9–16.3)	22.4 (19.4–25.4)	0.029
Flu positive	16.7 (13.9–19.4)	34.4 (28.1–40.6)	0.016
Flu negative	6.65 (4.88–8.40)	10.1 (8.69–11.6)	0.014

Peter Lougheed Centre			
	ROST	Standard	p value
Percent with oseltamivir prescription^a^			
Any result	24.2 (18.2–31.5)	34.2 (27.7–41.6)	0.054
Flu positive	81.8 (61.5–92.7)	73.1 (53.9–86.3)	0.52
Flu negative	14.9 (9.87–21.9)	27.5 (21.0–35.2)	0.0135
Mean number of doses^b^			
Any result	5.16 (4.51–5.80)	5.53 (5.12–5.95)	0.63
Flu positive	7.72 (6.73–8.68)	8.47 (7.67–9.28)	0.55
Flu negative	2.85 (2.39–3.32)	4.17 (3.85–4.49)	0.022
Mean hours from admission to first dose^b^			
All results	15.4 (12.5–18.2)	28.4 (19.1–37.8)	0.19
Flu positive	14.6 (10.1–19.2)	22.3 (17.2–27.4)	0.27
Flu negative	16.0 (12.3–19.7)	31.3 (17.8–44.8)	0.28

Data are in proportion(%) (95% CI) or mean (standard error of the mean). ROST= Rapid on-site testing

Alberta Children's Hospital: All results: ROST n = 181, standard n = 194. Flu positive: ROST n = 22, standard n = 25. RSV positive: ROST n = 40, standard n = 38. Flu and RSV negative: ROST n = 119, standard n = 131

Peter Lougheed Centre: All results: ROST n = 157, standard n = 175. Flu positive: ROST n = 22, standard n = 26. RSV positive: ROST n = 11, standard n = 9. Flu and RSV negative: ROST n = 124, standard n = 140

^a^Statistical analysis done using Fisher’s exact test

^b^Statistical analysis done using unpaired t-test assuming Gaussian distribution with two-tailed p value

#### Test turnaround time

Influenza A/B and RSV results on individuals randomized to ROST were reported in a median 1.41 h and 1.15 h compared to 27.7 h and 29.2 h (p < 0.0001) in the standard group for ACH and PLC, respectively (Table 3). The mean ROST TAT at ACH was also higher compared to PLC (2.49 h vs 1.39 h, respectively). During the same period in the last 2 years the median RPP TAT was 28.7 h (95% CI 28.9–32.2) and 30.2 h (95% CI 28.2–29.4) for ACH and PLC, respectively.

**Table 3:** Median time in hours from collection to first result

	ROST	Standard	p value
ACH	1.41 h (0.847–10.6)	27.7 h (11.7–60.8)	< 0.0001
PLC	1.15 h (0.762–2.82)	29.2 h (19.4–54.7)	< 0.0001

Data are in median (95% confidence intervals). Includes all admissions during the study (emergency department patients and inpatients) and excludes repeat tests on the same individual. Total samples from ACH study: ROST n = 322, standard n = 384. Total samples from PLC study: ROST n = 200, standard n = 222. Statistics performed using Mann–Whitney U test

#### Chest radiographs

For ACH inpatients there was a significant reduction in the percentage of patients with a CXR(s) ordered for ACH patients randomized to the ROST arm compared to the standard arm (17.1% vs. 28.9%, p = 0.0073, respectively) (Table 4). In the subgroup analysis, this reduction was only statistically significant in the influenza and RSV negative group. No statistically significant difference in CXR ordering was observed in the PLC study. There was a low proportion of CXRs done on ED patients (< 5% for both sites) and no significant difference was detected between arms regardless of test results (Additional file 1: Table S5). With respect to timing of CXR orders and ROST results, the majority of CXR orders for ACH and PLC inpatients (61.3% and 94.7%, respectively) occurred after the ROST result was reported (Additional file 1: Table S6).

**Table 4:** Percentage of inpatients receiving a chest radiograph (CXR), antibiotics or number of laboratory tests ordered

Alberta Children’s Hospital			
	ROST	Standard	p value
Percent with CXR order^a^			
All results	17.1 (12.3–23.3)	28.9 (23.0–35.6)	0.0073
Flu positive	9.09 (1.62–27.8)	16.0 (6.40–34.7)	0.67
RSV positive	22.5 (12.3–37.5)	36.8 (23.4–52.7)	0.22
Flu & RSV negative	16.8 (11.2–24.5)	29.0 (21.9–37.3)	0.025
Mean number of laboratory tests ordered^b^			
All results	3.85 (2.94–4.75)	5.39 (4.10–6.69)	0.057
Flu positive	2.41 (0.769–4.05)	2.64 (0.960–4.32)	0.84
RSV positive	2.23 (1.08–3.37)	4.00 (1.84–6.16)	0.14
Flu & RSV negative	4.66 (3.38–5.94)	6.32 (4.54–8.10)	0.14
Percent with an antibiotic order^b^			
All results	38.1 (31.4–45.4)	47.4 (40.5–54.4)	0.076
Flu positive	22.7 (10.1–43.4)	44.0 (26.7–62.9)	0.22
RSV positive	37.5 (24.2–53.0)	57.9 (42.2–72.1)	0.11
Flu & RSV negative	41.2 (32.7–50.2)	45.0 (36.8–53.6)	0.61

Peter Lougheed Centre			
	ROST	Standard	p value
Percent with CXR order^a^			
All results	24.4 (18.3–31.7)	26.9 (20.9–33.9)	0.61
Flu positive	4.55 (0.233–21.8)	26.9 (13.7–46.1)	0.055
RSV positive	36.4 (15.2–64.6)	22.2 (3.95–54.7)	0.64
Flu & RSV negative	26.8 (19.8–35.3)	27.1 (20.5–35.1)	> 0.99
Mean number of laboratory tests ordered^b^			
All results	5.33 (4.59–6.06)	6.51 (5.64–7.38)	0.043
Flu positive	5.09 (3.93–6.25)	7.85 (5.50–10.2)	0.046
RSV positive	6.64 (2.53–10.8)	7.00 (1.51–12.5)	0.90
Flu & RSV positive	5.25 (4.39–6.11)	6.23 (5.27–7.18)	0.14
Percent with an antibiotic order^b^			
All results	56.7 (48.9–64.2)	59.4 (52.0–66.4)	0.66
Flu positive	54.5 (34.7–73.1)	69.2 (50.0–83.5)	0.37
RSV positive	63.6 (35.4–84.8)	66.7 (35.4–87.9)	> 0.99
Flu & RSV negative	56.5 (47.7–64.9)	57.1 (48.9–65.0)	> 0.99

Data are in proportion (%) (95% confidence intervals) or mean (standard error of the mean). ROST=rapid on-site testing

Alberta Children's Hospital: All results: ROST n = 181, standard n = 194. Flu positive: ROST n = 22, standard n = 25. RSV positive: ROST n = 40, standard n = 38. Flu and RSV negative: ROST n = 119, standard n = 131

Peter Lougheed Centre: All results: ROST n = 157, standard n = 175. Flu positive: ROST n = 22, standard n = 26. RSV positive: ROST n = 11, standard n = 9. Flu and RSV negative: ROST n = 124, standard n = 140

^a^Statistical analysis done using Fisher’s exact test

^b^Statistical analysis done using unpaired t-test assuming Gaussian distribution with two-tailed p value

#### Laboratory test utilization

The utilization of laboratory tests was also affected by ROST. There was a statistically significant reduction in the average number of laboratory tests ordered between the ROST and standard arms in PLC inpatients (5.33 vs. 6.51, p = 0.043, respectively) with sub-group analysis showing a significant reduction in the influenza positive patients (5.09 vs. 7.85, p = 0.046, respectively). There were fewer laboratory tests ordered in the ACH inpatients for all results (mean 3.85 vs. 5.39 p = 0.057), regardless of influenza or RSV status, however this did not reach statistical significance (Table 4). There was no statistically significant reduction for either PLC or ACH ED patients between ROST and standard arms (Additional file 1: Table S5).

#### Antibiotic prescriptions

There was no statistically significant reduction in antibiotic prescription between the ROST and standard arms in inpatients at either ACH and PLC including subgroup analysis (Table 4) nor in ED patients (Additional file 1: Table S5). The majority of antimicrobial prescriptions occurred after the ROST result was reported: 67.0% and 76.3% of prescriptions for ACH and PLC inpatients, respectively (Additional file 1: Table S7).

#### Adverse events

There were no adverse events reported to the study team because of this trial.

## Discussion

Our pragmatic RCT is an important contribution to the literature on the clinical impact of rapid respiratory viral testing for four major reasons: (1) we are the first RCT to show a reduction in ancillary studies in both adult and paediatric populations, (2) we assessed multiple clinical and health care utilization outcomes in both paediatric and adult populations, (3) assessed these outcomes in these populations in a randomized manner, and (4) we generated results within the context and confines of complex health and accredited laboratory systems, thus representing the outcomes that would be seen when formally implemented.

The primary outcome for PLC (improved oseltamivir utilization) was met, but not for ACH (reduction in LOS). At both study sites there was a significant reduction in oseltamivir prescriptions and number of doses administered to influenza negative patients in the ROST arm compared to the standard arm, concordant with other adult and paediatric studies [17, 18], including two RCTs [8, 9]. We found a reduced mean time to first dose of oseltamivir in the ROST arm for patients with influenza A or B. This reached statistical significance at the ACH site with mean time to first dose being 18 h shorter for the ROST arm, thereby increasing the chance of patients receiving oseltamivir within the ideal 48 h from symptom onset window [19].

A recent RCT in adults has similarly demonstrated a decreased time to first dose [8]. Within the paediatric population, this has been previously shown in a retrospective cohort study in paediatric inpatients, but not a paediatric RCT [20]. Detecting this significant difference in the paediatric arm was unexpected based on our power calculations and due to increased oseltamivir prescriptions compared to prior years. This may have been due to knowledge of the trial causing higher oseltamivir prescriptions, due to ongoing quality improvement programs around respiratory disease (especially bronchiolitis), and/or a noted improvement in guideline adherence for appropriate oseltamivir usage within the paediatric population [21].

In contrast to several studies showing increased antiviral prescription in influenza positive patients [8, 9, 18, 20], we did not find a significant difference in oseltamivir prescription in influenza positive patients. Although this may have been due to insufficient power in this subgroup, our findings support the need for education on appropriate prescribing.

We found no significant difference in LOS between the ROST and standard arm at either ACH or PLC sites. Although there was an indication that LOS in paediatric inpatients may be decreased, our study was underpowered to adequately assess this outcome. The impact of rapid respiratory tests on LOS varies in the literature with before/after designs in paediatric populations showing either shorter admission durations [22] or no significance difference [18]. Similarly, results vary within the adult population. A non-blinded RCT by Andrews et al., found no difference in LOS [23], however due to delays in specimen processing their rapid test TAT was 19 h. Recently, a quasi-RCT (randomizing by week) by Bouzid et al., comparing POCT and central testing with Qiagen QIAstat-Dx Respiratory Panel V2® failed to show reduction in LOS with a rapid testing TAT of 1.1 h [16]. Likewise, no change to admission duration in adult inpatients was seen in a recent RCT by Clark et al., comparing  the Biofire Filmarray® respiratory panel 2  with a TAT of 1.2 h to standard of care (on-site PCR) [8]. In contrast, Brendish et al., in a parallel-group, open-label RCT with research staff performing testing, found a significantly shorter mean length of admission in adult inpatients tested with the Biofire Filmarray® respiratory panel group compared to conventional laboratory polymerase chain reaction (PCR) [9]. Other studies showing significant reduction in LOS are limited to specific populations or require multivariate analysis to adjust for clinical variables [22, 24, 25]. We were unable to ascertain differences based on clinical variables given no chart reviews were performed.

Regarding our secondary outcomes, we found a significantly shorter TAT in the ROST arm with results reported in a mean 26 h or more before the standard arm. Significantly reduced TAT with rapid testing is a consistent finding in rapid/ROST studies of various designs and quality [5, 8, 16, 20, 24, 26]. However, the optimal time for a respiratory NAT result has not been defined and is important to identify as it would guide which tests and locations of testing can lead to clinical benefit. An important consideration is surge capacity. ACH staff had noted increased testing during evening shifts related to surges in ED visits which negatively impacted ROST TAT (higher than PLC). Ensuring consistency in TAT is challenging as it may require additional investment in instruments and staffing.

Our RCT is the first pragmatic RCT to show a decrease in CXR orders associated with ROST. In the ACH study, we found a significant decrease in CXR orders in the ROST arm which has been shown previously in non-RCTs in both adult [27] and paediatric populations [20]. Of these orders, the majority occurred after ROST was resulted. Only one RCT showed a decrease in CXR orders, however they had intervened following rapid molecular testing to review the impact of the results with the ordering physician [14]. In our subgroup analysis the proportion of patients with a CXR ordered was lower in the ROST arm for all subgroups, but only statistically significant in the influenza and RSV negative group, perhaps due to insufficient power in the positive subgroups. Our local guidance is consistent with the AAP in that chest radiographs are not indicated for work-up of uncomplicated bronchiolitis [28, 29]. Our findings support the need for an ongoing quality improvement initiative led by paediatric physician leaders to decrease unnecessary CXRs at ACH.

ROST was also associated with improved laboratory test utilization, with a significant reduction seen in the subgroup analysis for PLC inpatients testing positive for influenza. This is an important finding, given previous studies (non-RCTs) have shown no significant difference in laboratory test utilization [11, 18]. Two non-RCTs which analysed subsets of diagnostic tests found significantly less orders post-rapid molecular implementation for ancillary microbiology testing [26] and C-reactive protein, complete blood count, and electrolyte analysis [27] in the rapid molecular cohorts. We did not differentiate between specific laboratory tests, however this would be important for future economic analysis.

Finally, we found no significant difference between antibiotic prescriptions between the ROST and standard arm at either site. This is consistent with other published randomized trials in adult [8, 9, 13, 16] and paediatric [13] populations, in addition to non-randomized trials in paediatric [18, 22] and adult [27] populations. Of note, at ACH the majority of those testing positive for a viral pathogen were prescribed antimicrobials after the result. This highlights that rapid test results in and of themselves are unlikely to change antimicrobial prescribing practice [14]. Antibiotic stewardship interventions within the ED and hospital settings need to be developed and supported in accordance with guidelines that suggest against antimicrobial therapy for viral respiratory illness [3, 29].

Overall, the impact of the ROST in our blinded study may have been reduced as physicians did not know the randomization until the result was reported. In a randomized study by Bonner et al., where the rapid influenza testing results were known prior to clinician evaluation of the patient, rapid testing reduced antibiotic use, laboratory testing, and radiographs [14]. An alternative pragmatic design would be alerting physicians of randomization during the test ordering process, thereby potentially leading to a greater impact on diagnostic and therapeutic decisions. This may have minimized the amount of tests ordered before the ROST result was reported. Our data showed the majority of oseltamivir prescriptions, CXR orders, and antimicrobial prescriptions occurred after the ROST result, but there is a lot of opportunity for improvement. The most obvious need for education is not prescribing oseltamivir after influenza negative ROST results were reported. This was true at both sites in all patient types, but most notably at PLC where 80% of ROST influenza negative patients were prescribed oseltamivir. The ROST could have greater impact on outcomes with educational programs addressing the trial design, appropriate ancillary testing, and treatment in accordance with guidelines [3, 29]. Future studies need to assess the impact of the outcomes when one or many stewardship interventions are implemented.

Prior studies have implemented rapid testing within the ED. We selected hospital laboratory testing over the ED due to practical and logistical considerations along with staffing constraints (i.e., training, quality management, availability of ED staff, space for GeneXpert installation, biosafety considerations, and reporting in LIS). Previously we have discussed implementing rapid testing within the ED, however it was deemed unfeasible. Also, due to the proximity of the lab to the ED and the wards, it was not necessary to implement in the ED to achieve a quick TAT.

The main limitation of our study was the lack of prospective recording of symptom onset date, diagnosis, and co-morbidities precluding the ability to control for these factors and perform associated subanalysis. Due to operational limitations and assay procurement regulations, we were unable to continue our study long enough to enrol the intended number of participants for the ACH primary outcome. In terms of randomization, although the pattern of rapid results could be ascertained by ordering clinicians, this was thought to be mitigated by the large size of these emergency departments and hospitals with the cycling of multiple care providers with various shift schedules. Additionally, our study did not address if rapid testing for pathogens other than influenza A/B or RSV on the standard of care panel would impact clinical management. Finally, we did not account for patients with tests positive for multiple targets. These patients only occurred in the ACH ED patients and were excluded only from subanalysis. Multiple targets should be considered a priori.

We did not assess isolation measurements for several reasons. Isolation days are not systemically recorded therefore recording this would require prospective monitoring which was beyond our funding. Additionally, per our Infection Prevention and Control Program, droplet isolation is based on symptoms and specific respiratory pathogen test result would only impact isolation if cohorting was required. This is compared to other regions where isolation to single rooms is based on positive respiratory pathogen testing [8, 16].

Although our enrolment period was partially eclipsed by the introduction by SARS-CoV-2 into Calgary, there was minimal impact given the low case number.

## Conclusions

Our findings justify implementation of rapid respiratory virus testing, which considering the emerging new landscape of respiratory pathogens since the SARS-CoV-2 pandemic, is more important than ever. Given the operational success of this trial, it has laid the foundation for the successful widespread implementation of rapid COVID-19, influenza, and RSV testing in our province at all urban hospitals. A rapid TAT with improved antiviral and diagnostic test utilization can offset the increased costs associated with these tests. An economic evaluation was beyond the scope of this study, but a dedicated study exploring this in detail should be performed. Furthermore, future studies should focus on clinical outcomes, identifying clinically relevant TAT and the impact of rapid testing for respiratory pathogens other than influenza or RSV.

## Supplementary Information

**Additional file 1: Table S1.** Validation of nasopharyngeal aspirates tested on the Xpert® Xpress Flu/RSV compared to standard nucleic acid testing. **Table S2.** Summary of discrepant results for ACH and PLC studies. **Table S3.** Oseltamivir utilization for emergency department patients. **Table S4.** Percent oseltamivir ordered before and after the ROST result was reported by virus detected. **Table S5.** Percentage of emergency department patients receiving a chest radiograph, antibiotics or number of laboratory tests ordered. **Table S6.** Percent chest radiographs ordered before and after the ROST result was reported by virus detected. **Table S7.** Percent antimicrobial ordered before and after the ROST result was reported by virus detected. **Figure S1.** Influenza A, influenza B, and RSV cases by Flu week for 2019–2020 for Calgary Health Zone. **Figure S2.** Survival curves for time to discharge of patients.

## Data Availability

The datasets used and/or analysed during the current study are available from the corresponding author on reasonable request. This study was retrospectively registered with ISRCTN, number 10110119. Study protocol is available as a supplementary file to this manuscript online or through the corresponding author.

## Abbreviations

ACH: Alberta Children’s Hospital
CXR: Chest radiographs
ED: Emergency department
IP: Inpatients
LOS: Length of stay
NAT: Nucleic acid test
NP: Nasopharyngeal
NPA: Nasopharyngeal aspirate
PCR: Polymerase chain reaction
PLC: Peter Lougheed Centre
RCT: Randomized controlled trial
ROST: Rapid on-site test
RPP: Respiratory pathogen panel
RSV: Respiratory syncytial virus
TAT: Turnaround times
UTM: Universal transport media
