# Supplementary material for: A pragmatic randomized controlled trial of rapid on-site influenza and respiratory syncytial virus PCR testing in paediatric and adult populations
Source: BMC Infect Dis. 2022 Nov 16;22:854. doi: 10.1186/s12879-022-07796-3 (PMC9667852; doi:10.1186/s12879-022-07796-3)
Supplement: Supplementary file 1 — Additional file 1: Table S1. Validation of nasopharyngeal aspirates tested on the Xpert® Xpress Flu/RSV compared to standard nucleic acid testing. Table S2. Summary of discrepant results for ACH and PLC studies. Table S3. Oseltamivir utilization for emergency department patients. Table S4. Percent oseltamivir ordered before and after the ROST result was reported by virus detected. Table S5. Percentage of emergency department patients receiving a chest radiograph, antibiotics or number of laboratory tests ordered. Table S6. Percent chest radiographs ordered before and after the ROST result was reported by virus detected. Table S7. Percent antimicrobial ordered before and after the ROST result was reported by virus detected. Figure S1. Influenza A, influenza B, and RSV cases by Flu week for 2019–2020 for Calgary Health Zone. Figure S2. Survival curves for time to discharge of patients. [file 12879_2022_7796_MOESM1_ESM.docx]

**Supplementary Material**

**Supplementary Methods**

*Pre-study testing*

Prior to the study, testing for respiratory viruses was done using the NxTAG^®^ Respiratory Pathogen Panel (RPP) (Luminex ^®^, Austin, TX) at a central laboratory in daily batches. At the clinician’s request, centralized rapid testing with Simplexa^®^ Flu A/B & RSV Kit (DiaSorin, Cypress, CA) was performed. Rapid testing was requested for 11% of respiratory virus NAT orders at PLC and 40% at ACH (August 2016-2017) with a mean TAT of 9.3 hours.

*Xpert^®^ Xpress Flu/RSV testing Nasal Pharyngeal Aspirate Validation*

Prior to the start of the study the use of raw or spiked NP aspirates previously confirmed negative for influenza A, influenza B, and RSV was validated for Xpert^®^ Xpress Flu/RSV testing since this sample type was not approved by Health Canada or the United States of America Food and Drug Administration (FDA) for this method. Specimens used for this validation were previously tested with the RPP or the United States Centers for Disease Control (CDC) Influenza A/B PCR by the Alberta Public Health Laboratory.[1] Primary NP specimens were used to spike the NP aspirates. Samples included 10 influenza A (n=5 pH1N1, n=5 H3N2), 9 influenza B (n=7 Victoria, n=3 Yamagata), 1 influenza A and B (H3N2 and Victoria), and 12 RSV (n=3 RSV-A, n=8 RSV-B, n=1 RSV-A and RSV-B positive), and 12 negative samples. Negative samples included samples negative for all targets on the RPP or positive for one or multiple of adenovirus, human metapneumovirus, enterovirus, parainfluenza 2, parainfluenza 3, and bocavirus. Extraction was performed using the Hamilton Microlab STAR (Hamilton Co., Reno, NV) with the Maxwell^®^ HT Viral Total Nucleic Acid kit (Promega Corp., Madison, WI). Results are found in supplementary table 1.

*Analytical performance of Xpert^®^ Xpress Flu/RSV and Lumiex NxTAG^®^ Respiratory Pathogen Panel*

Previously Xpert^®^ Xpress Flu/RSV has been shown in a recent systemic review and meta-analysis to have a sensitivity for Influenza A, Influenza B, and RSV of 97%, 98%, and 96% respectively, and a specificity of 100%, 100% and 97% , respectively [2]. The Lumiex NxTAG^®^ Respiratory Pathogen Panel has been shown to have sensitivity for for Influenza A, Influenza B, and RSV of 100%, 100%, and 100% respectively, and a specificity of 100%, 100% and 97.5% , respectively when compared to reference standards of Biofire Filmarray^®^ Respiratory Panel and singleplex real-time PCR [3].

*Ancillary laboratory tests*

Haemoglobin (alone or in panels such as complete blood count), electrolyte panel (sodium, potassium, chloride, bicarbonate), blood gas panels (venous, capillary, or arterial), cerebral spinal fluid cell count, urinalysis, blood cultures, *Bordetella pertussis* NAT, antistreptolysin O titre, mononucleosis test, Epstein-Barr virus (EBV) serology (EBV IgM, viral capsid antigen IgG, Epstein-barr nuclear antigen-1 IgG), cytomegalovirus IgG/IgM, and *Mycoplasma pneumoniae* IgM. Each test was counted as an individual test Each type of test was counted as one individual order (tests with multiple orders were counted more than once).

**Supplementary Tables**

Supplementary Table 1. Validation of nasopharyngeal aspirates tested on the Xpert® Xpress Flu/RSV compared to standard nucleic acid testing.

|  | Xpert Result | |
| --- | --- | --- |
| Reference Standard Result | Positive | Negative |
| Influenza A | 10 | 1 ^a^ |
| Influenza B | 9 | 1^b^ |
| Respiratory Syncytial Virus (RSV) | 11^c^ | 0 |
| Influenza A/B or RSV Negative | 0 | 12 |

Reference standard for influenza was the U.S. CDC influenza PCR^15^ and for RSV, the Luminex Respiratory Pathogen Panel (XTAG or NxTAG). One sample was positive for influenza A and B by CDC assay and Xpert. ^a^Ct value on the U.S. CDC assay was 36.19. ^b^Ct value on the CDC assay was 34.19. ^c^One sample had an error on the first run and was repeated. One sample that was positive for RSV-A and RSV-B had a probe check error, but insufficient sample was remaining to repeat and the sample was excluded from the analysis.

Supplementary Table 2. Summary of discrepant results for ACH and PLC studies.

| Standard arm result | | ROST arm result | | | | | |
| --- | --- | --- | --- | --- | --- | --- | --- |
|  |  | FluA | | FluB | | RSV | |
|  |  | Pos | Neg | Pos | Neg | Pos | Neg |
| FluA | Pos |  | ACH = 0  PLC = 1 (IND^a^, I/P^b^) |  |  |  |  |
|  | Neg | ACH = 1 (I/P)  PLC =1 (I/P) |  |  |  |  |  |
| FluB | Pos |  |  |  | ACH = 0  PLC = 0 |  |  |
|  | Neg |  |  | ACH = 1 (I/P)  PLC = 0 |  |  |  |
| RSV | Pos |  |  |  |  |  | ACH = 7 (6 I/P; 1 ED^c^)  PLC = 1 (I/P) |
|  | Neg |  |  |  |  | ACH = 0  PLC = 0 |  |

^a^IND = indeterminate result.  ^b^I/P = inpatient. ^c^ED = Emergency department patient

Note: For ACH patients that were positive by standard test (RPP), 3 of 6 I/P were positive for another virus (2 for human metapneumovirus and 1 for parainfluenza 4) and 1 of 1 ED patients (also positive for influenza A on RPP and Xpert). For the other discrepant results, no other virus was detected by the RPP.

Supplementary Table 3. Oseltamivir utilization for emergency department patients.

Supplementary Table 3A: Alberta Children’s Hospital

|  | ROST | Standard | p-value |
| --- | --- | --- | --- |
| Any result | 2.84 (1.11-7.07) | 0 (0-1.98) | 0.032 |
| Flu positive | 4.76 (0.85-15.8) | 0 (0-7.14) | 0.21 |
| Flu negative | 2.04 (0.363-7.14) | 0 (0-2.67) | 0.17 |

Data are in % (95% CI). All results: ROST n=141, standard n=190. Flu positive: ROST n=42, standard n=50. Flu negative: ROST n=140, standard n=98. There were four prescriptions for oseltamivir all in the ROST arm (2 influenza positive and 2 influenza negative). Among the PLC ED patients, Statistical analysis done using Fisher’s exact test.

Supplementary Table 3B: Peter Lougheed Centre

|  | ROST | Standard | p-value |
| --- | --- | --- | --- |
| Any result | 13.6 (6.40-26.7) | 14.9 (7.41-27.7) | 0.86 |
| Flu positive | 27.3 (9.75-56.7) | 27.3 (9.75-56.7) | >0.99 |
| Flu negative | 9.09 (3.14-23.6) | 11.1 (4.41-25.3) | 0.78 |

Data are in % (95% CI). All results: ROST n=44, standard n=47. Flu positive: ROST n=11, standard n=11. Flu negative: ROST n=33, standard n=36. There were 6 prescriptions for oseltamivir in the ROST arm (3 influenza positive, 4 influenza negative) and 6 in the standard arm (3 influenza positive, 3 influenza negative). One Flu negative patient in the ROST arm and one in the standard arm did not get a dose of oseltamivir in the ED. Statistical analysis done using Fisher’s exact test.

Supplementary Table 4: Percent oseltamivir ordered before and after the ROST result was reported by virus detected

Supplementary Table 4A: Alberta Children’s Hospital

|  | ROST Result | | |
| --- | --- | --- | --- |
| ED | All results | Flu positive | Flu negative |
| # oseltamivir ordered before ROST result | 2 | 0 | 2 |
| # oseltamivir ordered after ROST result | 2 | 2 | 0 |
| Total | 4 | 2 | 2 |
| % ordered after | 50.0% | 100.0% | 0.0% |
| I/P |  |  |  |
| # oseltamivir ordered before ROST result | 8 | 4 | 4 |
| # seltamivir ordered after ROST result | 13 | 11 | 2 |
| Total | 21 | 15 | 6 |
| % ordered after result | 61.9% | 73.3% | 33.3% |

Supplementary Table 4B: Peter Lougheed Centre

|  | ROST Result | | |
| --- | --- | --- | --- |
| ED | All results | Flu positive | Flu negative |
| # oseltamivir ordered before ROST result | 3 | 2 | 1 |
| # oseltamivir ordered after ROST result | 2 | 1 | 1 |
| total | 5 | 3 | 2 |
| % ordered after result | 40.0% | 33.3% | 50.0% |
| I/P |  |  |  |
| # oseltamivir ordered before ROST result | 12 | 8 | 4 |
| # oseltamivir ordered after ROST result | 26 | 10 | 16 |
| total | 38 | 18 | 20 |
| % ordered after result | 68.4% | 55.6% | 80% |

Supplementary Table 5. Percentage of emergency department patients receiving a chest radiograph, antibiotics or number of laboratory tests ordered.

Supplementary Table 5A: Alberta Children’s Hospital

|  | ROST | Standard | p-value |
| --- | --- | --- | --- |
| Percent with CXR order^a^ |  |  |  |
| All results | 5..67 (2.90-10.8) | 4.74 (2.51-8.75) | 0.80 |
| Flu positive | 4.88 (0.867-16.1) | 4.26 (0.756-14.2) | >0.99 |
| RSV positive | 9.09 (0.466-37.7) | 4.55 (0.233-21.8) | >0.99 |
| Flu & RSV negative | 5.68 (2.45-12.6) | 5.09 (2.35-10.7) | >0.99 |
| Mean number of laboratory tests ordered^b^ |  |  |  |
| All Results | 0.42 (0.24-0.61) | 0.21 (0.10-0.33) | 0.050 |
| Flu positive | 0.48 (0.05-0.91) | 0.13 (0.01-0.24) | 0.10 |
| RSV positive | 0.18 (-0.09-0.45) | 0.27 (-0.1-0.66) | 0.75 |
| Flu & RSV negative | 0.35 (0.16-0.54) | 0.24 (0.07-0.40) | 0.37 |
| Percent with an antibiotic order^b^ |  |  |  |
| All results | 4.26 (1.97-8.97) | 6.42 (3.7-10.88) | 0.47 |
| Flu positive | 0 (0-8.38) | 2.13 (0.1-11.11) | >0.99 |
| RSV positive | 9.09 (0.47-37.7) | 0 (0-14.9) | 0.33 |
| Flu + RSV negative | 5.68 (2.45-12.6) | 9.32 (5.29-15.9) | 0.43 |

Data are in % (95% CI) or mean (Standard error the mean). All results: ROST n=141, standard n= 190. Flu positive: ROST n=41, standard n=47. RSV positive: ROST n=11, Standard n=22. Flu and RSV Negative: ROST n=88, Standard n=118. Note: four individuals were excluded from sub analysis because they were positive for multiple targets; Standard arm had one patient positive for Flu A, Flu B and RSV, and two patients positive for Flu A and RSV. ROST arm had one patient positive for Flu A and RSV on rapid respiratory pathogen panel (RPP) and tested positive for Flu A only on ROST. ^a^ Statistical analysis done using Fisher’s exact test. ^b^ Statistical analysis done using unpaired t-test assuming Guassian distribution with two-tailed p value.

Supplementary Table 5B: Peter Lougheed Centre

|  | ROST | Standard | p-value |
| --- | --- | --- | --- |
| Percent with CXR order^a^ |  |  |  |
| All Results | 4.55 (0.81-15.1) | 4.26 (0.76-14.3) | >0.99 |
| Flu positive | 0 (0-25.9) | 9.09 (0.47-37.7) | >0.99 |
| RSV positive | 33.3 (1.71-88.2) | 0 (0-82.2) | >0.99 |
| Flu & RSV negative | 9.09 (3.14-23.6) | 11.1 (4.41-25.3) | >0.99 |
| Mean number of laboratory tests ordered^b^ |  |  |  |
| All Results | 0.53 (0.2-0.87) | 0.30 (0.005-0.6) | 0.79 |
| Flu positive | 0.18 (-0.2-0.59) | 0.36 (-0.3-0.98) | >0.99 |
| RSV positive | 1.33 (-4.4-7.07) | 0 (0-0) | 0.50 |
| Flu & RSV negative | 0.59 (0.17-1.0) | 0.29 (-0.08-0.7) | 0.29 |
| Percent with an antibiotic order^b^ |  |  |  |
| All Results | 20.9 (11.4-35.2) | 17.0 (8.9-30.1) | 0.64 |
| Flu positive | 9.09 (0.47-37.7) | 9.09 (0.47-37.7) | >0.99 |
| RSV positive | 33.3 (1.7-88.2) | 0 (0-82.2) | >0.99 |
| Flu & RSV negative | 24.1 (12.2-42.1) | 20.6 (10.4-36.8) | 0.77 |

Data are in % (95% CI) or mean (Standard error the mean). All results: Standard n= 47, ROST n=44. Flu positive: Standard n=11, ROST n=11, RSV positive: Standard n=3, ROST n=2. Flu and RSV negative: Standard n=34 ROST n=30. ^a^ Statistical analysis done using Fisher’s exact test. ^b^ Statistical analysis done using unpaired t-test assuming Guassian distribution with two-tailed p value.

Supplementary Table 6: Percent chest radiographs ordered before and after the ROST result was reported by virus detected

Supplementary Table 6A: Alberta Children’s Hospital

|  | ROST | | | |
| --- | --- | --- | --- | --- |
| ED | All results | Flu positive | RSV positive | Negative |
| # CXR ordered before ROST result | 0 | 0 | 0 | 0 |
| # CXR ordered after ROST result | 8 | 0 | 0 | 0 |
| Total | 8 | 0 | 0 | 0 |
| % ordered after | 100.0% | 0.0% | 0.0% | 0.0% |
| I/P |  |  |  |  |
| # CXR ordered before ROST result | 12 | 1 | 4 | 7 |
| # CXR ordered after ROST result | 19 | 1 | 5 | 13 |
| Total | 31 | 2 | 9 | 20 |
| % ordered after | 61.3% | 50.0% | 55.6% | 65.0% |

Supplementary Table 6B: Peter Lougheed Centre

|  | ROST Results | | | |
| --- | --- | --- | --- | --- |
| ED | All results | Flu positive | RSV positive | Negative |
| # CXR ordered before ROST result | 0 | 0 | 0 | 0 |
| # CXR ordered after ROST result | 2 | 0 | 1 | 1 |
| Total | 2 | 0 | 1 | 1 |
| % ordered after | 100.0% | N/A | 100.0% | 100.0% |
| I/P |  |  |  |  |
| # CXR ordered before ROST result | 2 | 0 | 2 | 2 |
| # CXR ordered after ROST result | 36 | 0 | 0 | 0 |
| Total | 38 | 0 | 2 | 0 |
| % ordered after | 94.7% | 0.0% | 100.0% | 100.0% |

Supplementary Table 7: Percent antimicrobial ordered before and after the ROST result was reported by virus detected

Supplementary Table 7A: Alberta Children’s Hospital

|  | ROST Results | | | |
| --- | --- | --- | --- | --- |
| ED | All results | Flu positive | RSV positive | Negative |
| # antibiotics ordered before ROST result | 12 | 1 | 2 | 9 |
| # AB ordered after ROST result | 2 | 0 | 0 | 2 |
| Total | 14 | 1 | 2 | 11 |
| % ordered after | 14.3% | 0.0% | 0.0% | 18.2% |
| I/P |  |  |  |  |
| # antibiotics ordered before ROST result | 30 | 2 | 5 | 23 |
| # AB ordered after ROST result | 61 | 4 | 14 | 43 |
| Total | 91 | 6 | 19 | 66 |
| % ordered after | 67.0% | 66.7% | 73.7% | 65.2% |

Supplementary Table 7B: Peter Lougheed Centre

|  | ROST Results | | | |
| --- | --- | --- | --- | --- |
| ED | All results | Flu positive | RSV positive | Negative |
| # antibiotics ordered before ROST result | 6 | 1 | 1 | 4 |
| # antibiotics ordered after ROST result | 3 | 0 | 0 | 3 |
| Total | 9 | 1 | 1 | 7 |
| % ordered after | 33.3% | 0.0% | 0.0% | 42.9% |
| I/P |  |  |  |  |
| # antibiotics ordered before ROST result | 23 | 4 | 4 | 15 |
| # antibiotics ordered after ROST result | 74 | 10 | 3 | 61 |
| Total | 97 | 14 | 7 | 76 |
| % ordered after | 76.3% | 71.4% | 42.9% | 80.3% |

**Supplementary Figures**

Supplementary Figure 1. Influenza A, influenza B, and RSV cases by Flu week for 2019-2020 for Calgary Health Zone.


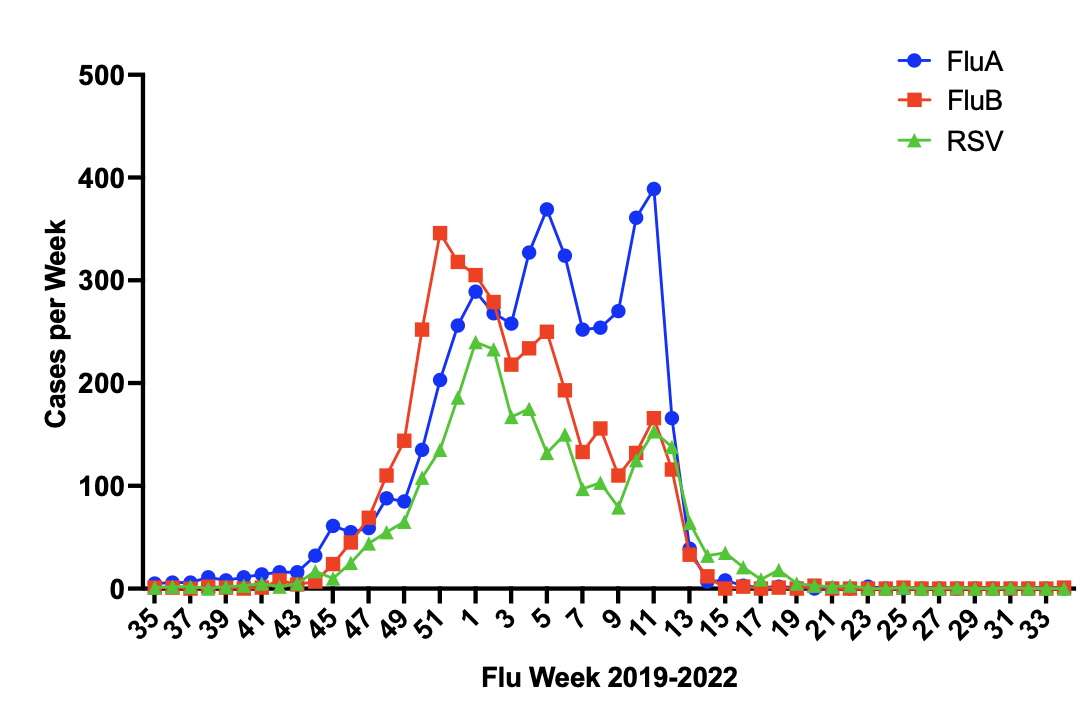
The study ran from Flu week 2 to week 11

Supplementary Figure 2. Survival curves for time to discharge of patients.

Legend: Hazard ratio (95% confidence intervals) for the ROST vs standard arms were as follows: ACH inpatients 1.22 (0.999-1.50), p=0.062; ACH emergency department 0.809 (0.651-1.01), p=0.056; PLC inpatients 1.17 (0.941-1.46), p=0.157; and PLC emergency department 0.806 (0.527-1.23), p=0.32.


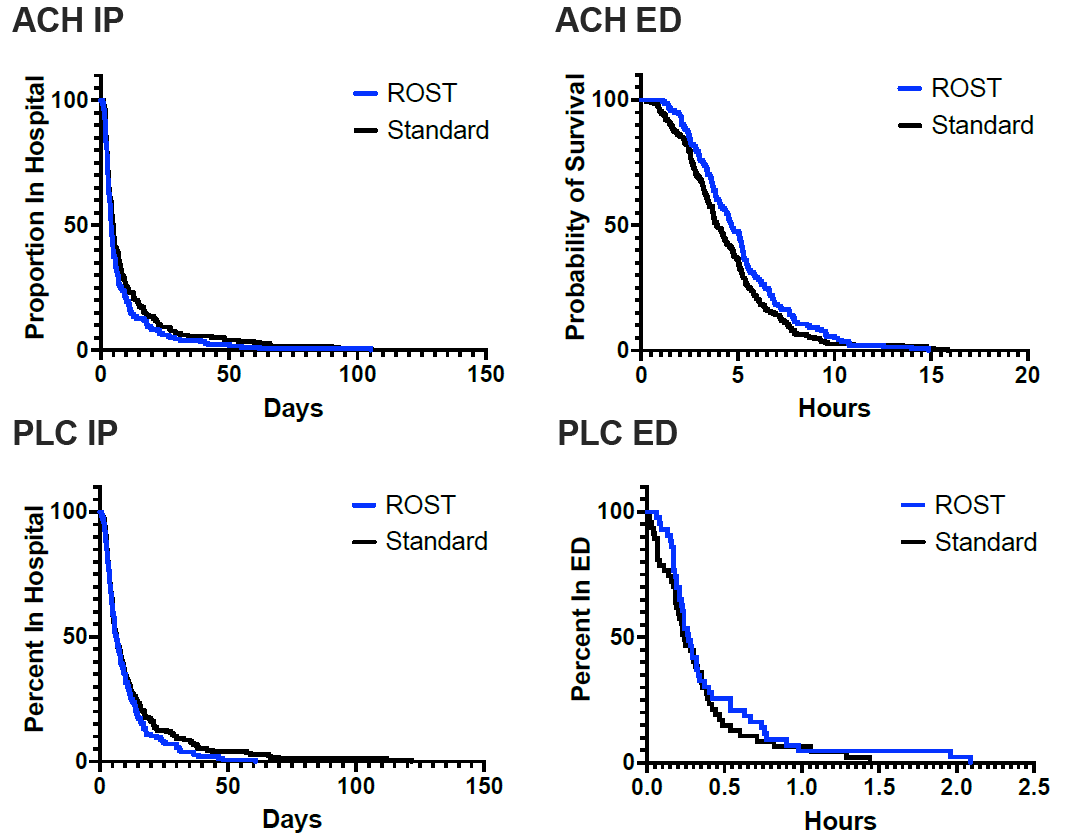


References

[1] Suresh BS, Rangaraj S. Evaluation of Three Influenza A and B Real-Time Reverse Transcription-PCR Assays and a New 2009 H1N1 Assay for Detection of Influenza Viruses. J Clin Microbiol 2010;48:3870–5. https://doi.org/10.1128/JCM.02464-09.

[2] Liu Y-L, Xie T-A, Lin G-L, Deng W, Lin Q-R, Pan Z-Y, et al. Diagnostic accuracy of Xpert Xpress Flu/RSV for detection of Influenza and Respiratory syncytial virus. Jpn J Infect Dis 2021. https://doi.org/10.7883/yoken.JJID.2020.987.

[3] Chen JHK, Lam H-Y, Yip CCY, Wong SCY, Chan JFW, Ma ESK, et al. Clinical Evaluation of the New High-Throughput Luminex NxTAG Respiratory Pathogen Panel Assay for Multiplex Respiratory Pathogen Detection. J Clin Microbiol 2016;54:1820–5. https://doi.org/10.1128/JCM.00517-16.
